# Methods in predictive techniques for mental health status on social media: a critical review

**DOI:** 10.1038/s41746-020-0233-7

**Published:** 2020-03-24

**Authors:** Stevie Chancellor, Munmun De Choudhury

**Affiliations:** 1 Department of Computer Science, Northwestern University, Evanston, IL USA; 2 School of Interactive Computing, Georgia Tech, Atlanta, GA 30308 USA

**Keywords:** Computer science, Medical research

## Abstract

Social media is now being used to model mental well-being, and for understanding health outcomes. Computer scientists are now using quantitative techniques to predict the presence of specific mental disorders and symptomatology, such as depression, suicidality, and anxiety. This research promises great benefits to monitoring efforts, diagnostics, and intervention design for these mental health statuses. Yet, there is no standardized process for evaluating the validity of this research and the methods adopted in the design of these studies. We conduct a systematic literature review of the state-of-the-art in predicting mental health status using social media data, focusing on characteristics of the study design, methods, and research design. We find 75 studies in this area published between 2013 and 2018. Our results outline the methods of data annotation for mental health status, data collection and quality management, pre-processing and feature selection, and model selection and verification. Despite growing interest in this field, we identify concerning trends around construct validity, and a lack of reflection in the methods used to operationalize and identify mental health status. We provide some recommendations to address these challenges, including a list of proposed reporting standards for publications and collaboration opportunities in this interdisciplinary space.

## Introduction

Researchers in computer science (CS) are using behavioral and linguistic cues from social media data to predict the presence of mood and psychosocial disorders. Since 2013, research can assess the presence of major depression^1–3^, suicidality^4–6^, eating disorders^7,8^, and schizophrenia^9^, among others with high accuracy (80–90%). In addition to mental disorders, these approaches are starting to assess related symptomatology, such as self-harm^8^, stress^10^, and the severity of mental illness^11^ without the use of in-person, clinical assessment. These signals are taken from the posting and behavioral history of social media websites and apps, such as Twitter, Reddit, and Facebook^12^. In this article, we adopt the term mental health status (MHS) to capture both mental disorders and these related symptomatology.

The benefits of these computational approaches to understanding MHS could be profound—for new data to supplement clinical care, assessing developing conditions, identifying risky behaviors, providing timely interventions, or reaching populations difficult to access through traditional clinical approaches. In fact, approaches like this have been adopted by platforms such as Facebook for suicide prevention efforts^13,14^. Complementary enthusiasm has surfaced in an emergent area known as “digital psychiatry”^15^, which leverages these predictive signals to improve mental health service outcomes.

In this new interdisciplinary space, there are few shared guidelines for what constitutes valid assessment of MHS in social media. Methods and insights for this work are drawn from interdisciplinary areas such as health informatics, machine learning, artificial intelligence, natural language processing, and human-computer interaction. Previous work in these domains has focused on abstract notions of ethics and methodological rigor to understand public health using social media data^16–19^. Reviews and meta-analyses have examined the expression of depression and anxiety in social media^20^; subjective mood, well-being, and mental health in social media^21,22^ and other non-clinical texts^23^; and the development of technology more broadly for mental and affective health^24–26^. Nevertheless, recent research has noted a lack of grounded recommendations detailing and evaluating current practices for building algorithms to predict MHS in social media data^16,27^.

Given the nascence of this field, we see incredible value in identifying trends in research methods and practices to identifying gaps before they systemically emerge in research paradigms. These issues are important not only as they reflect scholarly research quality, but also because, most importantly, the implications predicting MHS can have on individuals who may be the object of such predictions in clinical care and social media settings.

This article provides a critical review of methods in predicting MHS on social media, identifying 75 papers published between 2013 and 2018. We report on patterns of data annotation and collection, data bias management, pre-processing and feature selection, model selection, and validation. Our results reveal that there are issues in evaluating construct validity to determine and predict MHS that permeate the research process. We argue that this will inhibit reproducibility and extension of this work into practical and clinical domains, and we provide recommendations on how to begin to alleviate these problems.

## Corpus overview

**Fig. 1:**
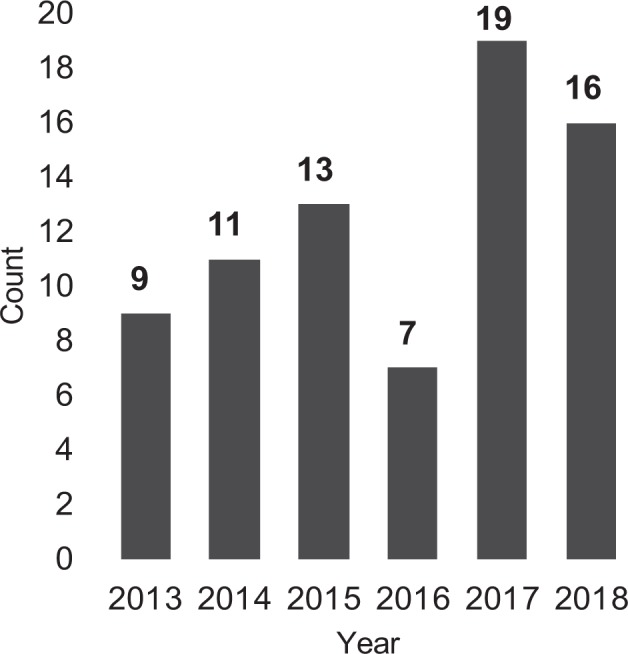
Publication counts by year. In this graph, we display the publication counts in our corpus from 2013 to 2018.

Figure 1 shows the years of activity in publication. The first research was published in 2013, with eight papers in total^1,28–34^. This area is showing rapid growth, with 19 papers in 2017^3,8,10,35–49^ and 16 in 2018^6,50–64^.

**Fig. 2:**
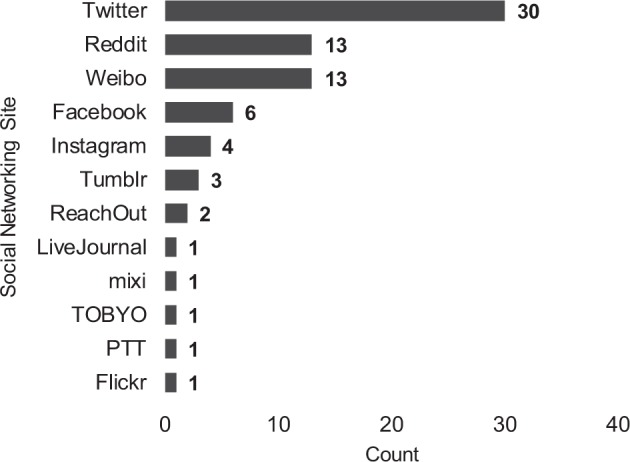
Publication counts by social networking site. In this graph, we display the counts of publications, organized by the various social networking sites studied.

We identified the social media platforms in these studies, summarized in Fig. 2. The most popular social media site for this analysis was Twitter, with a substantial portion (30/75) of the corpus studying this site (e.g. refs. ^65,66^). Other popular sites include Sina Weibo (13)^8,10,39,50,67–75^, Reddit (13)^6,41,46,48,52,54,55,58–60,63,64,76^, Facebook (6)^33,51,53,56,77,78^, Instagram (4)^3,11,38,62^, Tumblr (3)^7,44,79^, and ReachOut (2)^52,61^. Single papers inspect Flickr^8^, PTT^28^, mixi^29^, LiveJournal^80^, and TOBYO Toshoshitsu^81^. Year-over-year, Twitter was the dominant social media site examined in the corpus.

We also identified the representation of languages in publications. The majority of studies are done on English data (54) (e.g. ref. ^80^), followed by Chinese (14)^10,28,31,39,50,67–75^, Japanese (4)^2,29,32,81^, Spanish and Portuguese (1)^82^, and two that were not easily identified^38,47^.

### Disorders and symptomatology

**Fig. 3:**
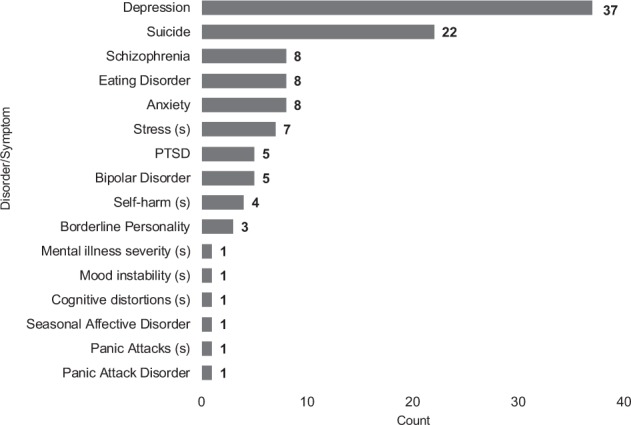
Publication counts by disorder and symptomatology. In this graph, we display the counts of publications that study specific disorders and symptomatology.

Next, we examined the disorders and symptomatology in each of the 75 papers. Eight papers studied more than one condition^36–38,48,65,83–85^, so we report the counts of unique disorders and symptomatology examined in Fig. 3.

Nearly half of the studies in the dataset (37/75) examined depression. Examples included studying depression generally^28,81,83,86^, major depressive disorder^1^, postpartum depression^30,78^, degree or severity of depression^77^, and depression as a risk factor for suicidality^39^.

We also found that 22 papers studied suicidality^4–6,29,36,39,48,49,59–61,66,68–70,72,76,80,87,88^. Cases included whether someone has suicidal ideation/is suicidal^29^, will attempt suicide^4,36,68^, or may shift to suicidal ideation in the future^76^. Other research looked at risk factors for suicide^39,87^, using crowdworkers for annotations of suicide risk^6^, and distinguishing between suicidal ideation and other discussions of suicide^5^.

Eight studies considered eating disorders^7,8,37,38,63,79,82,85^, most in the general case^8,37,38,63,82,85^, and two focusing on anorexia^7,79^. Another eight examined schizophrenia^9,36,37,43,48,59,85,89^. Lastly, eight studies used social media data to study anxiety, some in the context of other disorders^36,37,48,59,85^ and others more specifically^46,54,64^.

Other disorders and conditions investigated in our corpus included bipolar disorder (5)^37,48,65,83,84^, post-traumatic stress disorder (PTSD) (5)^37,83–85,90^, borderline personality disorder (3)^59,65,85^, and panic disorder (1)^37^.

Many studies also analyzed symptomatology related to mental disorders. This primarily focused on predicting stress (7/75)^10,39,41,67,71,73,75^. We also saw studies on self-harm (4)^48,52,59,91^, panic attacks (1)^36^, cognitive distortions (1)^44^, mood instability (1)^40^, and mental illness severity (1)^11^.

## Results

In this section, we summarize our findings about the corpus. Broadly, the field frames their study design and research investigations around prediction of mental illness from text and behavioral cues extracted from social media data. Almost all papers (69) conceptualized their research questions as a classification problem through binary classification (63/69), such as the categorical distinction between high and low stress^40^. Six papers used multi-class schema instead of binary classification^5,6,11,48,49,52^. Six papers used a model that predicts continuous or discrete values^32,38,53,69,77^. We also found that most studies (47/75) examine the individual/user as the object of prediction, such as predicting suicide risk of a person^39^. Twenty-five studies predicted mental health status per post or aggregated posts (e.g. refs. ^11,60^) and then, by proxy, inferring the MHS of the owner of those accounts. One paper examined both^42^.

To begin, in the absence of clinical assessment and in-person diagnosis, researchers have adopted alternative signals to identify positive and negative MHS. In this section, we examine questions of construct validity, or how the publications in the corpus validate the presence or absence of MHS.

### Establishing ground truth for positive annotation

We identified six methods of annotation for positive sign of MHS.

- Human Assessments (27). Many examinations asked humans to annotate the presence of MHS in a dataset. Domain experts, such as practicing clinicians or psychologists, were often called to annotate or label data^38,70^. For example, one study assessed depression through clinical interviews^31^. In other scenarios, CS researchers conducted the annotations^37,42^. Often, both domain experts and CS researchers partnered to annotate together^43,87^. Finally, some researchers used workers from crowdsourcing sites such as Amazon Mechanical Turk to identify status^5^ or verify the veracity of MHS downstream after another protocol^30^.
- Community or Network Affiliations (25). Researchers looked for community/network participation (e.g. refs. ^48,54^) to indicate MHS. Community participation was used as signal in social networks with formal communities, such as participating in communities about mental health on LiveJournal^80^, Reddit^41,46,48,59^, or posting in a suicide crisis community/forum^76^. These measures also included network signals such as following another account on Twitter^65,89^. Other studies use the signal of hashtags on apps like Instagram^11,38^.
- Self-Disclosure (17). This approaches searched for individuals to state that they suffer from a specific condition or are engaging in behaviors indicative of MHS^4,30,35,36,40,43,47,50,52,67,71,73,78,83,85,89,90^. These were triangulated with specific expressions, like “I was diagnosed with...”^83,90^. Positive annotation included stating that have a specific illness, like post-traumatic stress disorder^83^ or schizophrenia^43^. Work also examined self-reports of anti-depressant medication usage^35^, attempts to take their own life^4^, or self-described as being stressed or relaxed^67^.
- Administering Screening Questionnaires (14). Another popular technique was administering screening tools and questionnaires to consenting participants^1–3,32,33,39,45,51,53,62,66,69,72,77^. These included screeners that can measure depression, including the Center for Epidemiologic Studies Depression Scale (CES-D)^3,34,51^, Beck Depression Inventory (BDI)^1,2,33^, Patient Health Questionnaire (PHQ-9)^53,78^ and PHQ-8^62^, and Zung Self-Rating Depression Scale (SDS)^32^. Screeners were also used for other mental health status, such as suicidality^66,72^.
- Keyword use (10). Another approach used the presence of keywords or phrases^10,28,35,42,65,71,75,81,82,88^. Researchers used dictionaries connecting to suicide^88^ or stress^10,71^. Researchers also used symptom words and names of disorders on Twitter posts or profiles^42,82^, behaviors associated with disorders (like “ultimate goal weight”^8^), or if people use phrases associated with life events (e.g. childbirth)^30^.
- Acquired Annotations (9). Several publications acquired annotations from previously published research^31,37,50,57,84,86^ or shared tasks^49,52,61^.
- News Reports (2). Two studies looked at news reports of who had died by suicide to identify victims’ names, then find social media data on these individuals^68,70^.
- Medical Diagnostic Codes (1). One research study used the presence of the International Statistical Classification of Diseases and Related Health Problems 10th edition (ICD-10) codes from patient health records to detect depression^56^.

Some papers (33/75) took the results of the initial proxy assessments at face value (e.g. refs. ^41,46,80^). We noted that acquired datasets were often assumed to have high-quality labels, and the annotations were taken to be accurate^61^, as well as the use of screener questionnaires^45,51^. However, most studies (40/55) combined two approaches listed above to acquire a precise sample. Human annotation was a popular follow-up, with the validity of initial results of keyword matching often manually verified by researchers^54,65,88^. Other approaches used human verification to ensure that self-disclosure was genuine^9,42,43^. Two papers combined three ground truth assessment to triangulate MHS^4,66^. There was no reflection across the documents on what ground truth approach was appropriate for establishing construct validity, nor how many approaches combined together would accurately assess the MHS of interest. There was also no validation of applying constructs to social media data, for instance how strongly clinically valid screening questionnaires evaluate social media data.

## Source of control data/negative examples

Publications also sourced and design negative/control data for predictive tasks—these procedures were often different than the methods to identify positive signs of MHS.

- Validated No MHS (29). Many papers engineered ways to validate that the negative dataset did not contain people with the MHS of interest, e.g. refs. ^1,72,73^. This often was taking the lower bounds of cutoff from screening participants with screeners^2,33,51^. Other approaches used an expert to validate that there was an absence of MHS and no concerning symptoms disclosed in social media, such as no diagnosis of schizophrenia^43^.
- Random Selection of Control Users (23). Many studies constructed a negative/control dataset from randomly sampled data on the social media platform^4,6,8,9,29,35–37,41,43,46,47,54,59,68,70,79,81,83,85,89–91^. This random sampling came from historical samples of data, like the YFCC100m (Yahoo! Flickr Creative Commons 100 million) dataset^91^ or other collections^83^. Others gathered randomly, such as from random Tumblr users^79^ or the front page of Reddit^41,54^.
- Lack of Mental Health Disclosure (22). These studies used a lack or absence of disclosure of MHS as source for negative data^28,29,37,38,42,45,47,48,50,52,58,63–65,68,76,80,81,87,89^. Examples included sampling people who did not disclose having a condition^65,89^ or did not participate in communities related to mental health^28,80^.
- Matching Strategies (8). Some research took randomly sampled users and constructed matched samples along demographic/behavioral characteristics of the positively identified users^4,8,9,36,85,89^. This included matching on inferred traits, like age and gender^4,8,85^, engagement on the platform^52^, or time-matching controls^36^. One study matched controls on health information provided through electronic health records^56^.
- Acquired from Other Sources (3). Some research acquired datasets from alternative sources, boosting the size or scope of their dataset with other data^49,57,61^.

### Managing data quality and sampling strategies

Next, we report on our study of data quality, or how documents in our corpus curated the dataset for higher quality results. In our corpus, 53/75 studies filtered to manage issues of data bias or quality in their datasets:

- Platform Behavior Thresholds (28). Researchers described removing data for not meeting minimum content or engagement thresholds, e.g. refs. ^69,89^. This included behaviors such as having an account on the site of interest^1,78^. Most studies had minimum activity thresholds, such as a minimum number of posts^64,65,83^. Others looked for minimum friends/relationships^29^, engagement from others on a thread^61^, or platform engagement over time^29,36,52^.
- Legitimate Mental Health Mentions (17). These studies validated disclosures of MHS^4,5,7–9,11,34,38,41,76,78,82,83,87–89^ Some had strict thresholds on the precision of positive MHS^8,82^ or the time frame in which certain behaviors could occur^76^. For instance, one study looked for suicide attempts with discernible dates^4^. Others removed individuals for participating in eating disorder recovery communities, which confounded presence of an active eating disorder^7,11^.
- Restriction on Participant Characteristics (14). These studies excluded individuals based on certain characteristics or traits^1,3,33,36,39,40,45,51,53,62,66,72,73,88^, such as age^39,73^ or posts in English^51,62^. Other studies filtered participants on crowdsourcing sites based on overall approval ratings or a minimum number of previous tasks completed^3,45^.
- Quality Control During Online Surveys (7). Another threshold was removing participants for not passing quality control measures on the surveys, especially on surveys given through crowdsourcing sites such as Amazon Mechanical Turk or Crowdflower^3,33,34,51,66,69,72^. This included filtering surveys completed too fast^34,69^, who did not pass attention checks during the survey^3,66^, or did not finish the survey^33,51,72^.
- Removing Spurious Data (6). Other studies removed spurious data^39,66,72,81,88,89^, such as duplicate survey responses^39^ or gibberish^88^. One study mentioned removing advertisements^81^, and two removed spam^81,89^.

We did not notice any larger dataset adjustments to account for other kinds of biases, as noted by Olteanu et al.^92^ We inspected for whether studies adjusted for sampling bias or confounding factors with limited access APIs, adjusted for other clinically-relevant signals (such as demographics), took alternative data sampling strategies (such as selective rather than random sampling), or removed adversarial content, bots, or outlier/famous accounts (such as celebrities). Other than two papers that removed spam and advertisements^81,89^, we did not notice any corrections in the dataset for these factors. We also did not see larger analyses or adjustments to datasets to ensure that the samples were representative or accounted for population-level trends. The only management of these biases were in matching strategies to assemble negative datasets, e.g. refs. ^4,9,36^.

### Variable selection/feature engineering

Next, we examined patterns and characteristics of the data characteristics relevant for prediction. This is often referred to as variable selection or, in the machine learning community as “feature engineering”. In all, 42/75 studies reported the total number of features—of those 42 papers, the range of the number of features ranged from 7^11,29^ to over 15,000^76^.

- Language Features (68/75).
  - Structural/Syntactic (25). We found features that describe the structural or syntactic composition of social media posts, (e.g. refs. ^6,72,89^, such as the length of the post^39,76^, part-of-speech tagging^5^, and modality tagging^81^. We also saw counts of specific characters, like emoticons^89^. One study used the length and number of numeric characters in the domain name of a blogging site^72^.
  - Character and Word Models (38). These representations of language draw on probabilistic distributions of character and word patterns within text, e.g. refs. ^4,32^. This included $n$-gram use^87^, character modeling^68^, bag-of-words models^2^, term-frequency-inverse document frequency (TF-IDF)^28^, and word embeddings^38^. We also saw deep learning approaches to modeling language through convolutional neural networks^52^.
  - Topical (14). Other documents engineered features using topic modeling to identify meaningful connections between concepts in datasets^2,6,47,49,56,61,69,70,77,83,84,86,87^. This included the popular Latent Dirichlet Allocation (LDA) topic model^84,86^, and Brown clustering^9^.
  - Linguistic Style (18). Some studies used considered linguistic style and content measures as features^1,6,9,30,34,40,42,43,45,49,59,73,76,78–80,83,91^. Research used style categories from the Linguistic Inquiry and Word Count (LIWC) dictionaries^34,80^. We also noticed the study of readability, coherence, and perplexity measures^9,42^, as well as subjectivity measures from MPQA^49^ and TextBlob^59^.
  - Domain-Specific (13). Studies designed domain-specific linguistic features to evaluate text documents^1,5,42,47,49,58,71,76,80,81,83,89,91^. This included constructing dictionaries or lexicons related to depression^42,58,72^, self-harm^91^, suicide^5^, and stress^71^. This also included assessing user-generated mood tags taken from LiveJournal^80^ as well as explicit mentions of medication^1,46^. One study designed features around the final sentence as an indicator of suicidality or intent^49^.
  - General Language Measures (18). Papers also described generic language measures^5,6,8–10,39,44,46,49–51,60,61,64,66,69,77,84^, such as the LIWC library in its entirety.
- Behavior (37/75).
  - Activity (35). Features also tracked behavioral activity of the individual, e.g. refs. ^33–35^. Posting frequencies were a source of interest^4^, including volume of posts^76^, posting rates^65^, and temporal distributions of posting history^28^. Studies also examined platform-specific features, like geo-tagged posts^33^.
  - Interaction (31). Interactions with others on the platform were another common feature source, e.g. refs. ^61,67,79^. This included uni-directional follower/followee relationships^47,89^ and bi-directional friendships^33^. Papers also examined community membership/affiliation or participation^8,46^, platform affordances like Twitter’s retweet/quote, mentions/replies features^65^, or participation in threads from others^61^. Some other studies examined group membership as a variable^51^.
  - Network (6). Studies analyzed the network or graph structures for an individual’s social network^1,10,29,35,73,74^, including clustering coefficients and homophily^29^, strong and weak ties^10^, and network size, density, and depth^1,35,73^.
  - Domain-Specific (8). In addition to general behavioral features, publications also engineered domain-specific activity measures^1,10,29,31,51,61,72,83^. These features focused on measuring posting between the night hours, quantified as the “insomnia index”^1^. Another paper examined suicide homophily, or the number of friends who had died by suicide^29^. One study used previous evaluations of well-being on a crisis site in the predictive features^61^.
- Emotion and Cognition (38/75).
  - Sentiment, Affect, and Valence (36). Many papers examined peoples’ expressed mood, sentiment, and intensity of emotion, e.g. refs. ^41,53,62^. This was measured with sentiment scoring mechanisms like ANEW^80^, LIWC^7,78^, LabMT^62^, TextBlob^60,61^, and VADER^36^. Other studies examined affect and intensity^30^, polarity of emotions on more complex scales^53^, or counted the positive and negative emoticons^8,73^.
  - Psycholinguistic (11). Researchers also use psycholinguistic evaluations of emotional status from language^7,10,40,43,45,53,67,72,79,80,83^, using categories of emotional speech (such as anger or anxiety in LIWC)^80,83^.
  - Domain-specific (4). Domain-specific applications of emotion and cognition measurements included measuring personality traits via Big 5^84^, behavior theories of anorexia recovery^7^, a lexicon of emotional words related to mental distress^6^, and Tweets related to depression^42^.
- Demographic Features (11). Papers also incorporated data about personal demographics into variable selection^1,33,37,38,50,51,65,72,78,84^. This included age and gender^51,65,72^, education, income, and relationship status^1,47^. Some of these were not gathered from individuals in the dataset; rather, they were inferred using computational means^47,84^.
- Image Features (8). Researchers extracted visual information from the images of posts^3,10,38,47,50,67,75,91^. This included color themes/Hue-Saturation-Value (HSV) values^3,50,67^, if the image includes a face^3^, brightness and saturation values^10,47^, and the types of colors used^47,75^. This also included data extracted from a convolutional analysis of the images^38,91^.

For feature reduction or selection techniques, 26/75 described reducing features to salient ones, such as^5,39,82^ The most popular feature reduction technique was dimensionality reduction through Principal Component Analysis (PCA)^77,89^. Other feature selection methods included experimentally removing features^42^, deep learning-based reductions through convolution or GRUs (Gated Recurrent Units)^52,58^, feature ablation^9^, stepwise regression^39^, and taking $k$-best features^43^.

### Algorithm selection

Nearly all papers frame their contributions as predicting MHS; in that vein, most documents choose algorithms from machine learning and statistical modeling, and highlight prediction results in their findings. Two papers chose their algorithms for their ability to assess correlations between features^33,53^. No papers used pseudo-causal or causal approaches to make claims.

There was high diversity in algorithm selection, of which 73/75 papers reported on their algorithm of choice. The most popular predictive algorithm was Support Vector Machines, used by 24 projects^1,2,6,8,9,28,30,34,39–42,51,54,55,60,68,70,79,79,81,86–89,93^. Fifteen studies used logistic regression^4,11,29,44,56,60,61,63,64,72,72,73,76,78,80,82^. Next was Random Forest at seven papers in the corpus^3,5,36,43,45,65,72^, and one who used a Rotation Forest (a boosted version of Random Forest)^5^. We also saw the use of decision trees (2)^35,66^, Naïve Bayes (2)^31,82^, and XGBoost^49^. Finally, we found the use of regression techniques for some studies (8)^7,33,53,62,69,77,90,90^. This included the use of linear regressions^62,69,77^, log-linear regression^83,90^, correlational analyses^33,53^, and survival analysis/Cox regression^7^.

Deep learning has been a more recent trend, with 14 papers using this technique^10,37,38,46–48,50,52,57–59,67,75,91^. Some papers used more straightforward deep neural networks^8,46,67^, some with additional convolutional layers^48^, or recurrent neural networks^58,59^. Other research adopted a multitask neural network to share information between prediction tasks^37,71^.

How were these algorithms selected for use? In all, 41/75 papers described their process for selecting their algorithm of choice. The vast majority of algorithms (30/41) were selected because they performed the best, e.g. refs. ^3,34,50^, experimentally chosen across several algorithmic options^34,42^. Other reasons offered were the suitability of the model to the research task, such as sharing knowledge between tasks^37^, stability of model training^52^, interpretable features for clinicians and other stakeholders^63,66^, or dropout impacting the use of standard regression techniques^7^. Others drew from theoretical and practical reasons to select their models^5^, such as the “no free lunch theorem”^44^.

### Validating algorithms and reporting performance

72/75 papers reported how they validated the models, the most popular of which was using $k$-fold cross validation. Fifty-four papers use this technique, with a $k$ ranging from 5^40^, 10^82^, 20^62^ to leave-one-out^39,66^. Another common technique (20/72) was holding out blind data as a test set and reporting performance^4,11,42,43,48,50,52,57–59,63,73,76,77,86–89,91^; held-out dataset size ranged from 10%^88^ to 30–40%^69,91^. Next were multiple experimental runs of the model (14/72)^1–3,10,30,34,45,47,50,51,60,67,72,79^, ranging from 5^45^ to 1000^79^ runs. Three studies used model fit measures to validate the fit of the model, such as deviance for regression fit^7,11,29^ and feature relevance or curation techniques like stepwise regression to prevent overfitting^29,32^.

Many papers combined multiple validation techniques, the most common was cross-validating their test data and reporting results on a held-out dataset^30,88^ or pairing cross-validation with multiple experimental runs^34,72^.

Finally, 70/75 papers reported performance in a way that can be evaluated and benchmarked by other research. The best performance tended to be measured on machine learning metrics such as accuracy^46,51,80^, precision and recall^45,86,89^, F1 (a harmonic between precision and recall)^52,76^, and area under the curve (AUC)^56,62^. We occasionally found the use of regression-oriented measures, such as root mean squared error (RMSE)^69^ and ${R}^{2}$^77^. We very rarely saw use of popular metrics from other domains, such as sensitivity, specificity (or false positive/negative rates), and positive and negative predictive value^37^—the machine-learning oriented metrics dominated reporting standards.

### Essential reporting of prediction technique details

Last, we studied the reporting of essential information required to reproduce a predictive algorithm, which are de facto minimum standards to evaluate an approach. We identified five crucial factors essential to running any regression model or machine learning approach. These are: the number of samples/data points, number of variables/features, the predictive approach (either a specific algorithm or regression type), a method for validation, and the metric used to evaluate performance. We then counted the number of papers that explicitly reported on these five items:

- 71/75 number of samples/data points.
- 42/75 number of variables/features.
- 73/75 algorithm or regression of choice.
- 72/75 at least one validation method.
- 70/75 explicit performance or fit metrics.

We noticed that the most commonly omitted variable was the dimensionality or number of variables in the feature/variable space. For those that omitted this information, studies would describe what features were being included (such as word embeddings representation of the social media posts, or language models built on top of the post content), yet not include the size or number of their feature vectors. In five papers, we had difficulty assessing the performance of the selected regression or classification algorithm because the authors included this information on poorly-labeled graphs or figures. It was not possible in these graphs to assess the precise performance or fit of the model to the data, and we were forced to estimate from bar charts’ bands of performance, i.e. (80–85% F1).

Finally, we studied the patterns of reporting for all minimum standards across the dataset. If each paper is examined for the presence of these five traits, only 32/75 papers, or 42%, successfully reported all five measures. If we examined for four of five criteria, 67/75 papers, or 89%, reported on at least four of five criteria.

## Discussion

Our results demonstrate the variety of study design, methods techniques, and reporting schema to understand mental health status (MHS) of individuals through their social media data. Despite these innovations in techniques, we noticed concerning trends around construct validity with the identification and prediction of MHS in our corpus. Shadish et al. define construct validity as “making inferences from the sampling particulars of a study to the higher-order constructs they represent”^94^—said another way, this type of experimental validity maps theoretical constructs of knowledge to the observed phenomenon within the dataset. The challenges of construct validity in observational social media research in particular has been recognized^92,95,96^. These issues of construct validity risks deviating from known clinical and diagnostic criteria for MHS that ultimately may limit the reproducibility and application of this research.

### Concerns around construct validity

In our dataset, there was limited explication on the theoretical/clinical grounding of the MHS of interest, beginning with clearly defining what mental health concern is being measured, and how it is operationalized within the research.

Specifically, many papers did not leverage established theories in clinical science or clinical psychology to establish or ground the status they investigated or specifically defined the construct itself. For example, five studies examine the concept of anxiety^36,37,46,48,54,59,64,85^, though none operationalize what they mean when they study this particular disorder. Anxiety as a concept is overloaded—it is a category of nervous disorders, symptomatology that can influence other mental disorders, a transient emotion that people experience, and lay usage referring to emotional states and/or traits of a person. We see similar patterns for the notion of depression—it is frequently and subtly implied that the authors are referring to major depressive disorder; yet, these definitions are rarely explicated.

More ambiguities arise when documents establish positive and negative sources of data for identifying examples to pass to a predictive system. In our Results, we identified numerous innovations in techniques for positively identifying MHS—from hashtag use, e.g. #depression), follower networks, and digital administration of screening questionnaires like CES-D to consenting participants. However, in the documents, we rarely see reflection or evaluation of whether the new technique may measure the construct of interest. For example, the use of hashtags is a unique way to identify discussions of depression, but does it accurately identify those who suffer from major depressive disorder or is it another group of people interested in the topic? For less precise measurements, such as mood or stress, hashtags may be a valuable signal, but their application to diagnostic-level criteria is as of yet untested. Similar ambiguities on evaluating negative or “control” datasets also appear, as few studies establish that the research team was able to identify a lack of MHS in their populations. Even in the case of clinically-grounded approaches such as screening questionnaires, the papers do not establish the strength of the relationships between screening for MHS and the variables of interest.

These unstable constructs permeate through the experimental design, data collection and designing and selecting models. Rarely is reflection or justification provided that explain the selection and reduction of variables/features, data bias corrections, or algorithm selection. We see this gap manifest in what is reported for validation of predictive algorithms—only 32 of 75 papers reported explicitly five minimum standards for reproducing these algorithms. Additionally, we saw very limited use of causal analysis approaches or techniques to establish stronger relationships between the variables on social media and the MHS of interest, such as controlling for confounding factors or adjusting for sampling biases.

These challenges with construct validity jeopardize the credibility of identifying MHS and the replication of these studies in the future. As Ernala et al. also found in their explorations of schizophrenia prediction on social media^27^, the operationalization of identifying MHS is not connected to theoretically or clinically rigorous definitions of mental health, nor is the new method of identification formally or causally validated. Without construct validity being established, it is hard to know if the studies in our corpus indeed measure MHS in ways that may be useful for other audiences, such as clinicians, or if they are in fact measuring something else. Ernala et al. also showed that it is possible that we are measuring a complementary population of those interested in mental illness, of which a subset will likely have diagnoses^27^. However, if the implications of the work are being framed for clinical audiences and adoption, there must be stronger validation of the constructs in the research to be applied to clinical practices.

For replication, imprecise reporting of study details, such as variable selection criteria, can cause inappropriate or erroneous conclusions to be drawn from the results. For those unfamiliar with machine learning but are interested in the potential of these approaches, these gaps in reporting standards can imply that undisclosed researcher discretion guided the decision-making process, when, in fact, there are guided ways to approach problem solving in machine learning and artificial intelligence.

These gaps and unstable constructs may limit clinical and public health adoption of social media predictions of MHS. Many papers in the corpus indicate in their Introductions the potential for social media to augment clinical intake or assessment, the active management of mental disorder, guiding interventions, or accessing hard-to-reach populations^16^. However, with unstable construct validity and unclear methods documentation, the techniques in these papers may not be adopted for these purposes, as clinicians may not believe the measures are reliable for their patient populations. This may limit their adoption into real-world treatment protocols and designs.

### Moving toward better practices in research

In light of these findings, we are hopeful that researchers can adopt practices that would facilitate better validity of their measures and correspondingly influence downstream adoption into clinical practice. There have been calls by researchers from within social media and health research to consider these factors^16,19,27^, as well as broader calls around operationalizing constructs and abstraction in machine learning^97^. Workshops and symposia across disciplinary boundaries are emerging, designed to support more collaborative rigorous practices within this new area

Several studies within our corpus had strong construct validity that may serve as models in the dataset for best practices. Construct validity necessitates connection to clinically or theoretically-grounded practices—so grounding how MHS in these areas is operationalized is very important. This could be done in several ways. First, researchers could draw on relevant literature from domains like clinical psychiatry and psychology to anchor their approach, as De Choudhury et al. clearly defined the clinical research on major depressive disorder, then assessed it via administering screeners (like CES-D) to participants^1^. Similarly, Eichstaedt et al. used ICD-10 codes for diagnosis to establish the presence of MHS, then asked participants consent to examine their Facebook data for signs of depression^56^. We also advocate for collaborations with domain experts to guide the operationalization process for MHS; domain insights and guidance would be brought into the explication of the clinical terms to the social media context. In another paper, Burnap et al. partner with an expert on suicidality to build a classifier that distinguishes between six kinds of Tweets about suicide, ranging from those indicating legitimate disclosures of suicidality to awareness campaigns^5^.

We encourage this new area of research to be mindful of reporting practices within papers to facilitate better replicability and scholarship. These issues may be caused in part because of the interdisciplinarity of the area and lower awareness around the adoption of predictive models in research domains without background in machine learning or statistical modeling^26^. We believe that the concerning reporting practices across the corpus can easily be rectified with better reporting standards for data collection, annotation of data, and statistical modeling. In that vein, in Table 1, we propose several reporting standards that could be adopted by the area to provide clarity. These extend beyond our minimum reporting requirements, and include opportunities for better reporting of positive and negative signs of MHS, data bias and sampling strategies, and feature selection. We also believe that better reporting standards will avoid potential traps in erroneous conclusions being drawn without sufficient evidence or risky causal language being used, strengthening the quality of the research from this emergent area. This list is not intended to be an all-encompassing proposal for the field; in fact, the field should work to establish practices and guidelines for effective use of machine learning and predictive techniques in this domain area beyond these ideas.

**Table 1:** Our recommendations for standards for reporting for methods and study design.

Proposed standards for study design and methods reporting	
Ground truth validation procedures for all data	Explicit number of features/variables
Data source (API, scraping, etc.)	Variable/feature reduction techniques
Bias mitigation and sampling strategies	Algorithm used in best-performing scenario
Number of data points/samples	Hyperparameter tuning procedures
Source of all features/variables	Validation metrics
Error analysis and explanation	Explicit performance evaluation measures

We also advocate for the establishment of practices and norms by this nascent field of research through stronger connections to the traditions of clinical psychiatry. Domain experts like clinical psychiatrists, researchers in medicine, social workers with experience in mental illness, and other experts have valuable knowledge to direct this research to be more rigorous and accurately assess the constructs we claim to measure. As the field moves towards generalizing these findings to new social media platform or new opportunities for practice, it is essential that psychometric, especially construct validity is carefully maintained throughout these practices. Looking towards complementary fields like mobile health^98,99^, bioinformatics^100^, these areas have prioritized critical inquiry and reflection into their practices and have brought in clinical collaborators on their projects. This may also mean drawing on the methods of other areas to establish better validity, such as experiments, controlled study designs, and randomized control trials. By working with domain experts and adopting practices from this space, the research will improve as it is better able to “measure what we think [the concepts] measure”^92^[p. 5].

In conclusion, we offered a critical analysis of the methods, study design, and results reporting in 75 papers that predict mental health status on social media data. Our review identified key areas of similarity and trends within the field around data annotation and bias, pre-processing and feature selection, and model selection and validation measures. We also uncovered gaps in reporting procedures for minimum standards for methods validation, and gaps in precision in identifying the mental health status of interest. We hope that this meta-review provides the field guidance on the methods of interest in this space and guides researchers towards better reporting standards to encourage more reproducible and replicable science in this important area.

## Method

Constructing a literature review corpus across disciplinary boundaries is challenging because of the methods of publication. Unlike other fields which rely on journals, the most common venues for publication in CS are conference proceedings. When we tested our initial search strategy through standard indexing services, journal entries were robustly indexed; yet there were large gaps in conferences known to be important in these subfields across professional organizations (e.g. AAAI, ACL, ACM, NIPS/NeurIPS, AMIA). Initial experiments with keyword searches through engines like Google Scholar yielded over 200,000 candidate papers, which is intractable for searching.

To manage these challenges, our search consisted of 41 hand-selected venues (both conferences and journals) that “seeded” our search. Then, we used search terms to filter for candidate papers in these venues. Finally, we sampled the references of candidates once to identify any missing research. We found 75 papers in total—more extensive details of our process are included in the Supplementary Information.

### Search strategy

Two sets of keywords were developed to search in pair-wise fashion: those for mental health and those for social media. For mental health, 16 terms were identified, related to generic terms for mental health and disorders, the most common mood and psychosocial disorders, and symptomatology (e.g. stress, psychosis). This was informed by prior work^20,21^ and the DSM-V^101^. For social media, we searched for eight terms, including general terms for social media as well as three popular social networks, Facebook, Twitter, and Instagram. A list of our keywords can be found in Table 2.

**Table 2:** Keywords for literature search.

Category	Keywords
Mental health (1)	mental health, mental disorder, mental wellness, suicide, psychosis, stress
	depression, anxiety, obsessive compulsive disorder, post-traumatic stress
	disorder, bipolar disorder, eating disorder, anorexia, bulimia, schizophrenia,
	borderline personality disorder
Social media (2)	social media, social network, social networking site, sns, facebook, twitter
	instagram, forum
Search term	(1) AND (2)

To overcome the challenges mentioned above about indexing, 41 English venues were identified that could publish research on predicting MHS using social media data. This included a large set of CS conference venues across many sub-areas, general interest journals, and proceedings in health informatics and data science. A full list of these venues can be found in the Supplementary Information, Table 3.

We used three different search engines to ensure robust coverage across these venues, given our above indexing concerns. We used the Association of Computing Machinery (ACM) Digital Library for ACM journals and conferences, Google Scholar using the Publish or Perish software^93^ for other conference publications, and Web of Science for journals. One venue (CLPsych) was not indexed correctly by any search engine, so we manually searched the proceedings for matching keywords in the title and abstract. Using these strategies, we identified 4420 manuscripts that matched our keyword pairs.

### Filtering strategy

The manuscripts were filtered to only include peer-reviewed, original, and archival studies published between 2008 and 2017, dovetailing with the emergence of academic research on social media^102^. Certain kinds of publications were excluded, as they did not conform to our standards for originality: meta and literature reviews, commentaries and opinions, case studies, shared tasks, and non-archived submissions to CS conferences. After deduplication and filtering, this resulted in 2344 manuscripts.

Next, we manually filtered by title and abstract, removing items obviously not relevant to mental health or social media. Examples of mismatches included other health conditions, such as cancer, and data sources like electronic health records. This screening of titles/abstracts resulted in 87 papers.

Finally, all 87 papers were read and fully screened with the following criteria for MHS:

1. They must address mental health in clinically specific ways. This meant studying a mood or psychosocial disorder (e.g. depression), given symptoms from the DSM-V^101^ about disorders (e.g. suicide), or the generalized severity of mental disorders (e.g. moderate vs. severe depression). We excluded papers about subjective mood, well-being, happiness, or general emotions not directly related to mental disorder diagnosis (e.g. angry or happy). We also excluded papers about mental disorders and conditions that are not mood or psychosocially oriented (e.g. ADHD, autism spectrum disorder)^101^.
2. The paper’s method must focus on quantitative prediction. This included regression analysis, machine learning, and time series analysis.
3. The paper must study social media data, which we define as websites or apps that allow users to post/maintain content and profiles and interact/develop social networks and communities with others around said content^12,92,102^. Current examples would be Facebook, Twitter, Reddit, and Tumblr. We excluded other digital data traces, such as search engines, SMS/texting datasets, and fitness or mood trackers—these areas represent important areas for exploration but were out of scope for our study.
4. The prediction must be made on an individual. If a paper made predictions on individuals that were then aggregated for another purpose, we included these in our analysis.

This process generated 44 papers for analysis. Finally, to comprehensively expand our dataset beyond our 41 venues, we conducted a snowball sampling of related papers to extend the corpus of these 44 papers, identified from the bibliographic details from the citations, detailed in the Supplementary Information. This process identified 11 new papers, in turn providing 55 papers for inclusion in the review. In September 2019, we updated the dataset to search for 2018 data. This process and snowball sample identified 20 new papers, bringing the total number of papers in our corpus to 75. A full list of the documents, and details of our data collection process, are included in the Supplementary Information, Table 1.

### Analysis technique

We developed a priori a rubric for analyzing the manuscripts that included both descriptive, quantitative, and qualitative criteria, influenced by prior work^20,21,92,103^ and our understandings of the research space. This rubric had over 100 items, including data collection methods and pre-processing strategies, accuracy and baseline thresholds, results reporting mechanisms, and the presence of commentary on certain study design choices and implications of the research. We also recorded qualitative notes for analytical insights and thematic observations. To test the robustness of our rubric, we randomly selected four manuscripts of our corpus to annotate before beginning. We adjusted the rubric for additional reporting categories based on the results of our trial annotation. The relevant portions of our rubric design can be found in the Supplementary Information, Table 2.

We then conducted a close reading of all 75 papers in our corpus, annotating the rubric and identifying corpus-wide trends. The entire dataset was read and coded twice by the first author to standardize the coding process, each time in a random order. We then met and discussed the emergent themes and findings, which constitute our analysis.

### Reporting summary

Further information on research design is available in the Nature Research Reporting Summary linked to this article.

## Supplementary information

Reporting Summary

Supplementary Information

## Data Availability

All data generated and papers analysed during this study are included in this published article (and its Supplementary Information files).
