# Supplementary material for: Methods in predictive techniques for mental health status on social media: a critical review
Source: NPJ Digit Med. 2020 Mar 24;3:43. doi: 10.1038/s41746-020-0233-7 (PMC7093465; doi:10.1038/s41746-020-0233-7)
Supplement: Supplementary file 2 — Supplementary Information [file 41746_2020_233_MOESM2_ESM.pdf]

Supplementary Materials

Supplementary Figures

We include a PRISMA diagram to illustrate our literature search process.

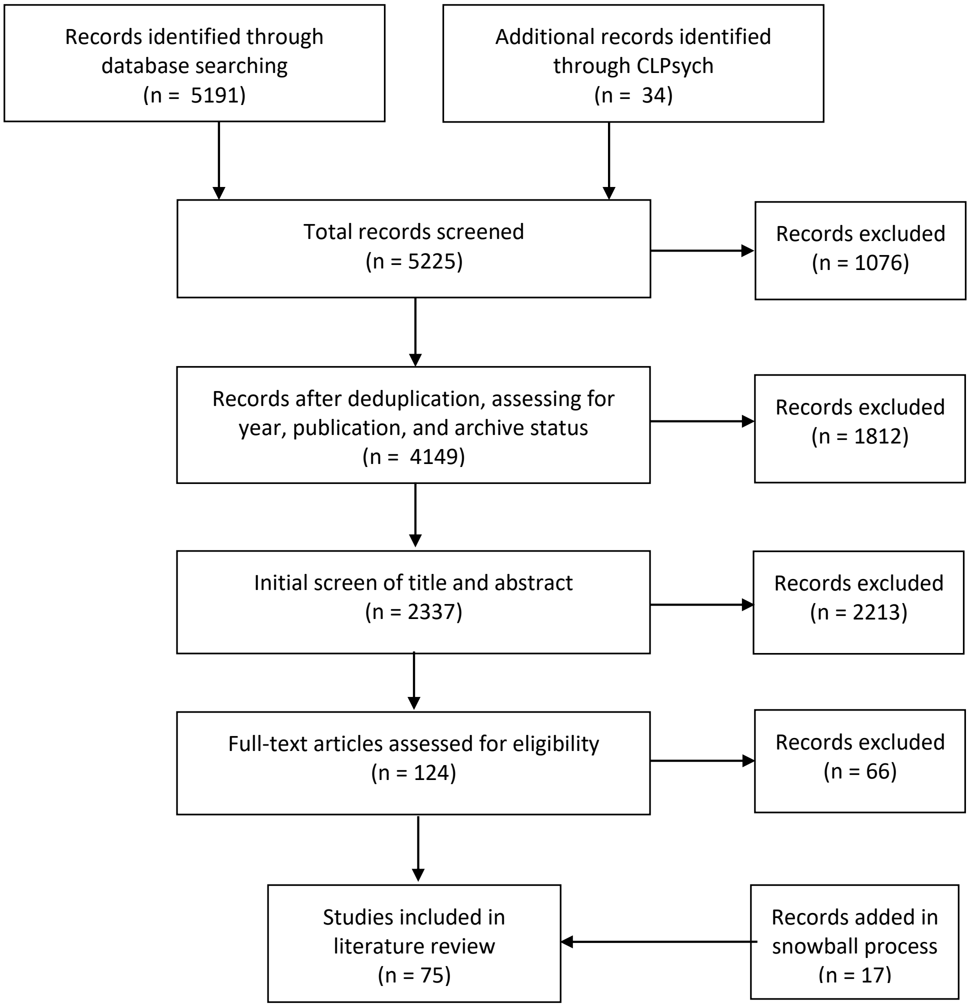

Figure 1. PRISMA Diagram of our corpus collection and compilation approach.

Supplementary Methods

Venues List

We selected 41 English language venues, given the constraints of the authors in understanding English. This includes CS conference proceedings across human computer interaction (e.g. CHI), social computing (CSCW), health informatics (DH), machine learning (NIPS/NeurIPS), computer vision (ECCV), artificial intelligence (AAAI), and natural language processing (ACL). We also include journals for general interest research (Nature, Science), medicine and medical informatics (Bmj), health and internet research (JMIR), and data science (EPJ Data Science). This includes venues across professional societies (ACM,

IEEE), the Association for Computational Linguistics (ACL), independent conferences (NeurIPS/NIPS, AMIA), and journals. These are displayed in Supplementary Table 1.

Iteration/Snowball Approach

We honored the respective standards for archival status within each home venue, given the diversity of venues. For example, workshop papers within SIGCHI are not considered archived, whereas within ACL they are if available in the online digital library.

Finally, we conducted a iterative pass, sampling related papers to our 44 identified from the bibliographic details of the citations. We identified 519 candidates; after deduplication, this produced 253 unique papers. After filtering for date and archive status, there were 200 left. After screening the title and abstract and de-duplicating these citations against our 44 entries, there were 20 unique papers. Finally, after a full paper screen, we identified 11 new papers for analysis. Additional snowballs through these papers did not return substantially new results.

2018 Update

In September 2019, we added additional data to cover publications released in 2018. For this procedure, we replicated the search criteria detailed in the Methods section and added an additional filter for the year to be 2018. We identified 804 entries after deduplication. After filtering for publications and archive status, there were 736. After screening the title, there were 125 candidates; then after screening the abstracts, there were 31 remaining. We read and fully screened the papers, and found 16 papers from 2018. To ensure fair coverage, we snowballed on these papers, just as before. The snowball process identified 82 candidate papers. Through our entire filtering process for snowballs, we found 4 papers in total.

Rubric Design and Testing

Our approach for analyzing these trends was part of a larger research project on conducting a systematic literature review on predicting mental health status in social media data. We designed a rubric to assist in annotating the corpus of 55 papers with over 100 items. To validate the rubric in being robust, we piloted it on 4 randomly selected papers and made adjustments to the categories of inclusion. In addition, to ensure robust coverage of trends, we encouraged note-taking to track insights while doing close readings. We report here on the rubric categories and annotation items that were referenced in our analysis.

Supplementary Tables

Complete List of Papers In Corpus

In Table 2, we present all 75 papers we included in our analysis, organized by social network of interest.

**Authors Year, Citation**	**Mental Illness Status**
Facebook	
De Choudhury et al 2014[78]	Post-partum depression
Eichstaedt et al 2018[56]	Depression
Park et al 2013[33]	Depression

Schwartz et al 2014[77]	Degree of depression
Wongkoblap et al 2018[51]	Depression
**Instagram**	
Chancellor et al 2016[11]	Mental illness severity
Reece and Danforth 2017[3]	Depression
Ricard et al 2018[62]	Depression
Zhou, Zhan, and Luo 2017[38]	Depression; eating disorders
**ReachOut**	
Cohan et al 2017[49]	Suicide crisis
Soldani et al 2018[61]	Suicide crisis de-escalation
**Reddit**	
Alada et al 2018[60]	Suicidality
Chancellor et al 2018[63]	Pro-eating disorder
De Choudhury et al 2016[76]	Suicidal ideation
Dutta et al 2018[54]	Anxiety
Gkotsis et al 2017[48]	Bipolar disorder; borderline personality disorder; schizophrenia; anxiety; depression; self harm; suicide crisis
Ireland and Iserman 2018[64]	Anxiety
Ive et al 2018[59]	Borderline personality disorder; bipolar disorder; schizophrenia; anxiety; depression; self harm; suicide crisis
Pirina and Çöltekin, Çağrı 2018	Depression
Sadeque et al 2018[58]	Depression
Saha and De Choudhury 2017[41]	High or low stress
Shen and Rudzicz 2017[46]	Anxiety
Shing et al 2018[6]	Suicide risk
Yates et al 2018[52]	Depression; self harm
**Sina Weibo**	
Cheng et al 2017[39]	5 risk factors for suicidality - suicide probability; Weibo suicide communication; depression; anxiety; stress levels
Guan et al 2015[72]	High suicide risk
Huang et al 2015[70]	Suicidal ideation
Huang et al 2014[68]	Suicidal ideation
Lin et al 2014[67]	Stress
Lin et al 2014[75]	Stress
Lin et al 2017[10]	Stress
Lin et al 2016[71]	Stress; stress item (What is causing stress)
Wang et al 2013[31]	Depression
Zhang et al 2015[69]	Suicide risk score (SPS value)
Shen et al 2018[50]	Depression
Wang et al 2013[31]	Depression
Zhao, Jia, and Feng 2015[73]	Stress
**Tumblr**	
Chancellor, Mitra, and De Choudhury 2016[7]	Recovery from anorexia
De Choudhury 2015[79]	Anorexia content; Anorexia versus in-recovery
Simms et al 2017[44]	Cognitive distortions
**Twitter**	
Benton, Mitchell, and Hovy 2017[19]	Non-neurotypical; anxiety; depression; suicide; eating disorder; panic attack; schizophrenia; bipolar disorder; post-traumatic stress disorder
Birnbaum et al 2017[43]	Schizophrenia
Braithwaite et al 2016[66]	Suicidal communication
Burnap, Colombo, and Scourfield 2015[5]	Suidical vs 5 other classes about suicide-related communication

Coppersmith, Dredze, and Harman 2014[83]	Bipolar disorder; depression; post-traumatic stress disorder; seasonal affective disorder
Coppersmith et al 2015[85]	Anxiety; bipolar disorder; borderline personality disorder; depression; eating disorder; obssesive compulsive disorder; post-traumatic stress disorder; schizophrenia; seasonal affective disorder
Coppersmith, Harman, and Dredze 2014[90]	Post-traumatic stress disorder
Coppersmith et al 2016[4]	Suicide Attempts
De Choudhury, Counts, and Horvitz 2013[30]	Post-partum changes
De Choudhury, Counts, and Horvitz 2013[34]	Depression
De Choudhury et al 2013[1]	Depression
Homan et al 2014[87]	Distress (related to suicide)
Jamil et al 2017[42]	Depression (both user and tweet level)
Loveys et al 2017[36]	Anxiety; eating disorder; schizophrenia; suicide attempt; panic attacks
McManus et al 2015[89]	Schizophrenia
Mitchell, Hollingshead, and Coppersmith 2015[9]	Schizophrenia
O'Dea et al 2015[88]	Suicide
Orabi et al 2018[57]	Depression
Preotiuc-Pietro et al 2015[84]	Depression; post-traumatic stress disorder
Prieto et al 2014[82]	Depression; eating disorders
Reece et al 2017[45]	Depression; post-traumatic stress disorder
Resnik et al 2015[86]	Depression
Saha et al 2017[40]	High or low mood instability
Saravia et al 2016[65]	Bipolar disorder; borderline personality disorders
Seabrook et al 2018[53]	Degree of Depression
Shen et al 2017[47]	Depression
Tsugawa et al 2015[2]	Depression
Tsugawa et al 2013[32]	Depression score (Zung Self-rating)
Vedula and Parthasarathy 2017[35]	Depression
Wang et al 2017[8]	Eating disorders
Other SNS	
Nakamura et al 2014[81]	Depressive symptoms [TOBYO Toshoshitsu]
Nguyen et al 2014[80]	Depression [LiveJournal]
Wang et al 2017[91]	Self-harm [Flickr]
Shen et al 2013[28]	Depressed vs. sad [PTT (Taiwanese Bulletin Board System)]
Masuda et al 2013[29]	Suicide Ideation [mixi (Japanese social network)]

Table 1. Full list of papers from corpus.

In Table 2, we overview our larger rubric for analyzing the data that is relevant to this study.

**Identifying Mental Health Status**	
Source of Ground Truth (e.g. self-report; external validation; battery; etc.)	Manual (Human at every level) vs Automated Labeling of Ground Truth
Validation of Ground Truth (2nd procedure to validate)	Diagnostic Level
Clinical Involvement In Assessment of ground truth label	Source of Negative/Control Data
**Study Design and Participant Recruitment**	
Public, private, or another kind of data access	Subject inclusion/exclusion criteria at initial recruitment
Engagement with Human Subjects (participatory, observational, etc.)	
**Data Gathering and Filtering**	

Unit of analysis (post, user, something else)	Data sampling strategy (given a dataset, how were examples chosen?)
Data Gathering Method (API, scraped, acquired, solicited, etc)	Pre-processing of data
Procedures to adjust the data stream/solve API issues	Removal of adversarial accounts (e.g bots, spam, ads, etc)
Language of data	Procedures for deliberate omission of data
Number of unique users in dataset	Procedures for missing data
Number of unique posts/equivalent measure	Filtering Criteria (before dataset is finalized, how is it filtered)
**Feature Engineering**	
Type of Data Analyzed	Social Interaction Features (Interactions with others; others' interactions with the person; retweets of others; replies)
Number of features	Activity Measures (Measure of engagement, volume of posts, time spent on platform)
Are features data-driven or grounded?	Clinical Features (self-reported meds; MIS)
Linguistic Features (Tfidf; LDA; BoW; word embeddings)	Psycholinguistic Features (LIWC; PA and NA; readability; valence and arousal)
Image Features (color; tone; pixel information; etc)	Demographic Features
Network Features (clustering, modularity, etc)	Other Features
Dimensionality Reduction or Feature Selection	
**Predictive Algorithm Setup and Results**	
Algorithm of choice	Hyperparameter Tuning
Causal language used	Implied experimental or hypothesis-driven conditions
Type of prediction (categorical; binary/multi; continuous)	Baseline assumptions
Prediction Task (What is the prediction explicitly)	Validation Methods (cross-val; check model fit; test on blind dataset)
Performance (on chosen, best metric)	Error Analysis
Reasons for algorithmic exclusion	Synthetic Sampling Procedures
Selection of performance metrics	

Table 2. Details of our rubric items used for this analysis.

In Table 3, we present the Topic Areas of Interest and selected conferences and journals.

Topic Area of Interest	Conferences and Journals
General Interest	Science, Nature, PLoS One, PNAS
Data Science and Data Mining	KDD, WebSci, WSDM, HT, WWW, MM, TOKDD, TWEB, EPJ Data Science
Health, Medicine, & Health Informatics	JAMA, DH, AMIA, PervasiveHealth, bmj, JMIR, JMIR Mental Health
HCI and Social Computing	CHI, CSCW/ PACM HCI, GROUP, ASONAM, SocInfo, TOCHI, ICHI
Natural Language Processing	ACL, EACL, NAACL, EMNLP, CLPsych
Machine Learning & Computer Vision	NIPS/NeurIPS, CVPR, ECCV, ICML, ICCV
Artificial Intelligence	AAAI, IJCAI
Other	ICWSM, UbiComp/IMWUT

Table 3. The venues to identify documents related to mental health and social media research
